# Dynamic changing smoking habits and cardiovascular events in patients newly diagnosed with hypertension, diabetes, or dyslipidemia: a national cohort study

**DOI:** 10.3389/fcvm.2023.1190227

**Published:** 2023-06-28

**Authors:** Shinjeong Song, Hye Ah Lee, Yeji Kim, Bo Kyung Jeon, Chang Mo Moon, Junbeom Park

**Affiliations:** ^1^ Department of Internal Medicine, College of Medicine, Ewha Womans University, Seoul, Republic of Korea; ^2^ Clinical Trial Center, Ewha Womans University Mokdong Hospital, Seoul, Republic of Korea

**Keywords:** smoking habits, cardiovascular risk, hypertension, diabetes mellitus, dyslipidemia

## Abstract

**Background and aims:**

This study aimed to examine the association between dynamic smoking habit change and cardiovascular risk in a population newly diagnosed with hypertension, diabetes, and dyslipidemia.

**Methods:**

This study included 49,320 individuals who had received health examinations provided by the Korea National Health Insurance Service. To determine the hazard ratios (HRs) and 95% confidence intervals (CIs) for incident major adverse cardiac events (MACE) and all-cause mortality based on dynamic smoking habit changes for 2 years, multivariable Cox proportional hazard models were utilized.

**Results:**

During the follow-up, there were 1,004 (2.2%), 3,483 (7.6%), and 334 (0.7%) cases of myocardial infarction, stroke events, and cardiovascular death, respectively. The group with worsening smoking habits had an increased risk of cardiovascular events and death (HR: 1.33, 95% CI: 1.26–1.40) compared to improved smoking habits. The robustness of the results determined by a series of sensitivity analyses further strengthened the main findings.

**Conclusions:**

Our findings suggest that worsening of smoking habits, even for a short period of time, may increase the risk of myocardial infarction, stroke, and cardiovascular death in patients diagnosed with hypertension, diabetes, and dyslipidemia. For the primary prevention of cardiovascular events in patients with underlying diseases, dynamic modification of smoking habits should be actively considered.

## Introduction

Cardiovascular diseases (CVDs) have a notable impact on global health, leading to high rates of illness and mortality. The inadequate adoption of preventive measures and suboptimal management of risk factors associated with atherosclerotic cardiovascular disease (ASCVD) significantly contribute to this burden among adults (1, 2). According to data from the World Health Organization, smoking accounts for 10% of all CVD cases (3). Smoking increases the risk of all-cause mortality and is a cause of ASCVD (4, 5). According to the guidelines on CVD prevention, smoking cessation is recommended as the 1st step and Class I regardless of the established ASCVD status and age (6, 7). Studies on CVD risk reduction after smoking cessation have been widely conducted in persistent smokers and former smokers (8–13).

CVD risk estimates among newly diagnosed patients with hypertension, diabetes, or dyslipidemia could be improved by dynamic changing smoking habits, as well as by an objective and time-updated assessment of other CVD risk factors. We evaluated the relationship between dynamic changes in smoking habits during two biennial screenings after the diagnosis of chronic conditions, such as diabetes, hypertension, and dyslipidemia, and subsequent CVD events using large, population-based data. The data comprised of records on sociodemographic factors, health surveys, examinations, and medical claims from the National Health Insurance Service (NHIS) in the Republic of Korea. Additionally, we explored whether this relationship varied by age, body mass index, and blood pressure.

## Materials and methods

### Study population

For this study, we used a database provided by the Korean NHIS, which is a mandatory and universal health insurance system that covers 97% of the South Korean population. The remaining 3% with the lowest income are covered by the Medical Aid Program. As the NHIS database contains information on medical aid subjects, it represents the entire Korean population. The NHIS provides biennial health screenings for all insured South Koreans, including blood tests, chest x-ray examinations, physical examinations, and lifestyle questionnaires. We utilized data from the National Health Insurance Service-Health Screening Cohort (NHIS-HEALS Cohort), a nationwide retrospective cohort spanning from 2002 to 2015, to conduct this study. Further information regarding the NHIS cohort design, methods, and the validity of records can be found in prior research (14).

**Figure 1:**
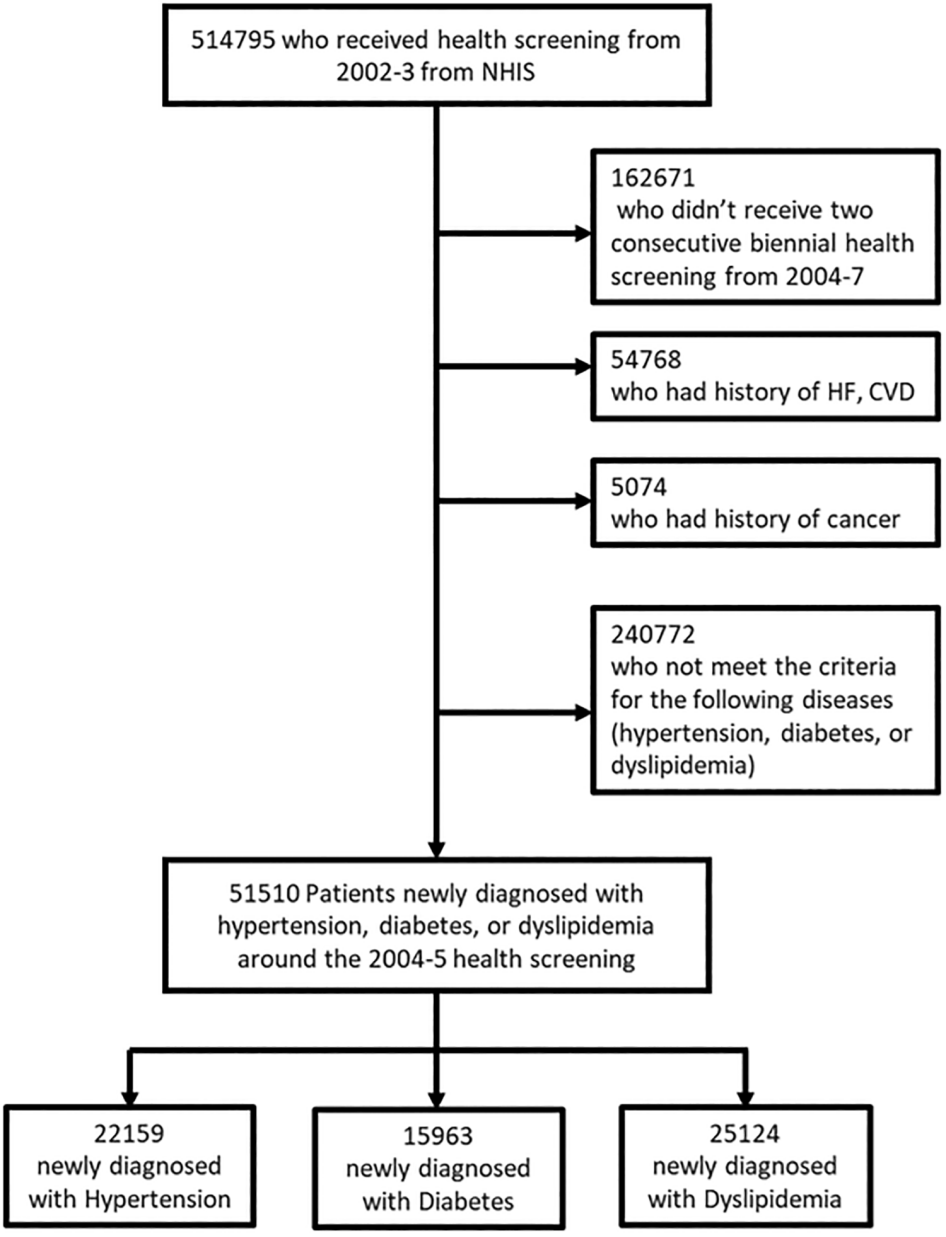
Flow diagram of the study population selection from the national health insurance service database.

The purpose of this study was to investigate the association between dynamic changes in smoking habits during a 4-year follow-up period (from 2002 to 3 to 2006–7) and CVD (myocardial infarction, stroke, cardiovascular death) in patients newly diagnosed with chronic diseases such as HTN, DM, or dyslipidemia. The NHIS-HEALS cohort enrolled 514,795 men and women who underwent national health screenings provided by the NHIS from 2002 to 2003. Among them, this study excluded participants without national health screening data from 2002 to 2007 and a history of heart failure or CVD (myocardial infarction, stroke) or cancer. Then, during the 4-year follow-up period, we included those with newly identified HTN, DM, or dyslipidemia. Finally, a population of 49,320 patients was included in the analysis (Figure 1).

The Institutional Review Board (IRB) at the Ewha Womans Medical College Mokdong Hospital (IRB no. EUMC-2021-11-029) and the NHIS Big Data Steering Department (NHIS-2022-2-197) approved this study. We were exempted from obtaining written consent from the participants because the NHIS data contains strictly anonymized clinical data that follow the guidelines of the Personal Data Protection Act.

### ICD 10 code definition

To assess behavioral changes in smoking before and after disease diagnosis, the incident diseases were confirmed for a period of ±1year from the date of the second health check-up. According to the International Classification of Diseases, 10th revision (ICD-10), newly diagnosed patients were defined as subjects who had 2 or more outpatient visits or at least 1 hospitalization with the diagnosis code for hypertension (ICD-10: I10-I15), diabetes (ICD-10: E10-E14), or dyslipidemia (ICD-10: E78) (15, 16).

We confirmed information on the medical claims records of the NHIS to identify CVD events during the follow-up period up to December 31, 2015. We used hospitalization records to identify the causes for all cardiovascular events (composite CVD events including myocardial infarct (MI) (ICD-10: I21), stroke (ICD-10: I60–I69), and cardiovascular death (I20–I25, I71–I72, and I60–I69)).

### Definition of smoking status

During each national health screening period, NHIS cohort participants completed self-administered questionnaires about their smoking habits and other lifestyle behaviors. Based on their responses from the first to third health check-up periods, participants in the biennial health checkup were categorized as current smokers or non-smokers. For the subgroup analysis, non-smokers at the time of examination were classified into never-smokers and past smokers according to whether they had a history of smoking. Accordingly, the smoking status investigated at the first health check-up was considered as the status before the disease diagnosis, and the smoking status at the third health check-up was regarded as the status after the disease diagnosis. Dynamic changes in smoking habits were defined as improved, worsened, or unchanged through the combination of smoking status at two-time points. Improvement in smoking habit was defined as quitting smoking, and worsening in smoking habit was defined as current smoking status from non-smoking or past smoking.

### Key variables

As covariates, age (continuous, years), income (continuous, quintiles), body mass index status (categorical, <23.0, 23.0–24.9, and ≥25.0 kg/m^2^), systolic blood pressure (continuous, mmHg), total cholesterol (continuous, mg/dl), fasting serum glucose (continuous, mg/dl), cigarette smoking (categorical, non-smoker and current smoker), alcohol consumption (categorical, 0, 1–2, 3–4, and ≥5 times per week), physical activity (categorical, 0, 1–2, 3–4, and ≥5 times per week), medication use (aspirin, antihypertensive medication, antidiabetic medication, and statin; categorical, yes or no), and Charlson Comorbidity Index (continuous) at the first check-up were considered. The Charlson Comorbidity Index is a method of categorizing the comorbidities of patients based on the ICD diagnosis codes found in administrative data, such as hospital abstract data. Each comorbidity category has an associated weight (from 1 to 6), based on the adjusted risk of mortality or resource use, and the sum of all the weights results in a single comorbidity score for a patient. A score of zero indicates that no comorbidities were found. The higher the score, the more likely the predicted outcome will result in mortality or higher resource us (16). Socioeconomic factors (age, sex, income) and medical factors (medication use and comorbidity; Charlson Comorbidity Index) were obtained from the NHIS insurance eligibility and medical claims databases, respectively (17). The annual income level was classified into five groups using quintile ratios (from Q1, the lowest, to Q5, the highest). Lifestyle behaviors (physical activity and alcohol consumption), laboratory results (total cholesterol, and fasting serum glucose), and measurement (body mass index, blood pressure) were determined from the NHIS national health screening database.

### Statistical analyses

The study participants’ characteristics at the beginning of the study were evaluated by expressing categorical variables as numbers and percentages and continuous variables as means and standard deviations. We determined the incidence rate (IR) by calculating the incidence per 10,000 person-years (PY) based on the number of total cardiovascular events and PY in each group, as per changes in smoking habits. The Cox proportional hazard model was used to calculate the hazard ratio (HR) and 95% confidence intervals (CIs) for CVD outcomes according to the dynamic changes in smoking habits between the two national health screening periods (2002–3 and 2006–7). In the Cox regression model, the following variables were adjusted for calculating the adjusted HRs and 95% CIs for CVD outcomes: age, income, body mass index, systolic blood pressure, total cholesterol, fasting serum glucose, cigarette smoking, alcohol consumption, physical activity, medication use (aspirin, antihypertensive medication, antidiabetic medication, statin), and comorbidity (Charlson Comorbidity Index). Multicollinearity was assessed using variance inflation factors and confirmed when the value was lower than 3. All data collection, mining, and statistical analyses were performed using SAS software (ver. 9.4; SAS Institute, Cary, NC, USA). The statistical significance was two-sided, and a *P*-value <0.05 was considered significant for all analyses.

## Results

### Baseline characteristics

Among the 49,320 participants, the mean (SD) age was 53.6 ± 8.9 years and 26,139 (53.0%) were men. About two-thirds of the individuals at the first (2002–3) and third (2006–7) health screening periods responded that they were never smokers (69.0% in the first and 69.7% in the third period, respectively). Other characteristics of the participants who underwent the two health screenings between 2002 and 2007 are listed in Table 1. Mean follow up duration was 10.9 years [interquartile range; 10.4–11.5yrs] after the screening in 2006–7.

**Table 1:** Characteristics of participants with a newly-diagnosed chronic disease (hypertension, diabetes, dyslipidemia) in the national health insurance service cohort who received two national health screening in 2002–3 and 2006–7.

Characteristics	All	Men in the NHIS cohort	Women in the NHIS cohort
Number of participants	49,320 (100%)	26,139 (53.0%)	23,181 (46.8%)
Smoking habit at screening period (2002–3)			
Never smoker	34,012 (69.0%)	11,587 (44.3%)	22,425 (96.7%)
Past smoker	4,535 (9.2%)	4,331 (16.6%)	204 (0.9%)
Current smoker	10,773 (21.8%)	10,221 (39.1%)	552 (2.4%)
Smoking habit at screening period (2006–7)			
Never smoker	33,267 (69.7%)	10,868 (43.8%)	22,399 (97.6%)
Past smoker	5,938 (12.4%)	5,819 (23.5%)	119 (0.5%)
Current smoker	8,538 (17.9%)	8,116 (32.7%)	422 (1.8%)
Age, mean ± SD	53.6 ± 8.9	52.7 ± 8.8	54.6 ± 8.8
Income status			
1Q	7,783 (15.1%)	3,195 (11.7%)	4,588 (19.0%)
2Q	7,155 (13.9%)	3,174 (11.6%)	3,981 (16.5%)
3Q	8,202 (15.9%)	4,283 (15.6%)	3,919 (16.2%)
4Q	10,898 (21.2%)	6,267 (22.9%)	4,631 (19.2%)
5Q	17,472 (33.9%)	10,462 (38.2%)	7,010 (29.1%)
Alcohol consumption			
None	28,385 (56.1%)	8,888 (32.9%)	19,497 (82.8%)
1–2 times/week	7,445 (14.7%)	4,970 (18.4%)	2,475 (10.5%)
3–4 times/week	8,494 (16.8%)	7,365 (27.2%)	1,129 (4.8%)
≥5 times/week	6,279 (12.4%)	5,830 (21.6%)	449 (1.9%)
Physical activity			
None	28,455 (56.9%)	12,917 (48.6%)	15,538 (66.4%)
1–2 times/week	11,918 (23.9%)	8,032 (30.2%)	3,886 (16.6%)
3–4 times/week	4,759 (9.5%)	2,998 (11.3%)	1,761 (7.5%)
≥5 times/week	4,849 (9.7%)	2,632 (9.9%)	2,217 (9.5%)
BMI (kg/m^2^), mean ± SD	24.5 ± 2.9	24.5 ± 2.8	24.5 ± 3.1
<23.0 kg/m^2^	15,825 (30.8%)	7,855 (28.7%)	7,970 (33.1%)
23.0–24.9 kg/m^2^	14,439 (28.1%)	7,887 (28.8%)	6,552 (27.2%)
≥25.0 kg/m^2^	21,196 (41.2%)	11,614 (42.5%)	9,582 (39.8%)
Total cholesterol (mg/dl), mean ± SD	206.4 ± 39.6	204.4 ± 39.6	208.7 ± 39.5
Fasting serum glucose (mg/dl), mean ± SD	101.2 ± 37.4	104.5 ± 40	97.5 ± 33.9
SBP (mmHg), mean ± SD	130.8 ± 18.2	132.7 ± 17.7	128.5 ± 18.5
DBP (mmHg), mean ± SD	81.9 ± 11.9	83.9 ± 11.7	79.7 ± 11.7
Newly diagnosed hypertension	22,180 (43.1%)	12,430 (45.4%)	9,750 (40.4%)
Newly diagnosed diabetes	15,983 (31.0%)	8,884 (32.5%)	7,099 (29.4%)
Newly diagnosed dyslipidemia	25,148 (48.8%)	12,644 (46.2%)	12,504 (51.8%)
Use of aspirin	2,222 (4.3%)	1,267 (4.6%)	955 (4.0%)
Charlson Comorbidity Index			
0	32,920 (63.9%)	18,735 (68.4%)	14,185 (58.8%)
1	12,802 (24.9%)	6,043 (22.1%)	6,759 (2.08%)
≥2	5,788 (11.2%)	2,603 (9.5%)	3,185 (13.2%)

### Dynamic change in smoking habits

Of the 34,012 participants who were never smokers at the first health screening period (2002–3), most of the participants were continuously never smokers (96.3%) and only 3.7% started smoking. About 36.6% of the current smokers at the first health screening period quit smoking after a diagnosis of chronic disease, and 91.2 percent of those with aggravated smoking habits were men. Compared to the first examination, at the second examination, 3,566 patients (7.5%) showed an improvement in smoking habits, 1,345 (2.8%) had aggravated habits, and 42,832 people had no change in smoking habits.

### Association of dynamic changes in smoking habits with total CVD

During 535,771 PY of follow-up, there were 5,021 total cardiovascular events, 1,122 myocardial infarctions, 3,927 stroke events, and 373 cardiovascular deaths. Smoking cessation was associated with a decline in myocardial infarction, stroke, and cardiovascular death vs. continued smoking (Table 2) in men. Similarly, cardiovascular events increased significantly during the follow-up period in the group with worsening smoking habits—new smoker or increasing the amount of smoking (adjusted HR (95% CI) myocardial infarct; 1.87 (1.35–2.59), stroke; 1.34 (1.09–1.65), cardiovascular death; 2.05 (1.19–3.55). However, there was no significant relationship between dynamic changes in smoking habits and cardiovascular events in women.

**Table 2:** Association of dynamic changes in smoking habit between the two biennial health screening periods (2002–3 and 2006–7) with subsequent risk of total cardiovascular disease events in the national health insurance service cohort.

Category	Number of participants	Cases	Incidence per 10,000 PY (95% CI)	Unadjusted hazard ratio (95% CI)	Multivariable-adjusted hazard ratio (95% CI)
Men					
Myocardial infarct	23,763	605	23.83 (22.00–25.80)		
Improving	3,319	89	25.09 (20.39–30.89)	1.09 (0.87–1.37)	0.81* (0.63–1.05)
Worsening	1,227	45	34.31 (25.62–45.96)	1.50 (1.10–2.03)	1.87** (1.35–2.59)
Stroke	23,763	1,800	72.20 (68.94–75.61)		
Improving	3,319	220	62.91 (55.12–71.79)	0.86 (0.74–0.99)	0.76* (0.65–0.89)
Worsening	1,227	102	79.20 (65.23–96.16)	1.08 (0.88–1.32)	1.34** (1.09–1.65)
Cardiovascular death	23,763	201	7.69 (6.70–8.83)		
Improving	3,319	19	5.20 (3.32–8.15)	0.66 (0.41–1.06)	0.49* (0.30–0.83)
Worsening	1,227	16	11.83 (7.25–19.31)	1.50 (0.90–2.51)	2.05** (1.19–3.55)
Women					
Myocardial infarct	22,106	399	16.89 (15.31–18.63)		
Improving	247	10	38.83 (20.89–72.17)	2.36 (1.26–4.42)	1.25 (0.49–3.17)
Worsening	118	3	24.37 (7.86–75.57)	1.48 (0.48–4.62)	0.91 (0.23–3.67)
Stroke	22,106	1,683	72.90 (69.50–76.47)		
Improving	247	29	114.98 (79.90–165.46)	1.61 (1.11–2.32)	1.14 (0.67–1.93)
Worsening	118	17	144.81 (90.02–232.93)	2.05 (1.27–3.30)	1.67** (1.03–2.69)
Cardiovascular death	22,106	133	5.54 (4.68–6.57)		
Improving	247	2	7.43 (1.86–29.72)	1.35 (0.33–5.43)	1.98 (0.18–21.89)
Worsening	118	1	7.80 (1.10–55.40)	1.41 (0.20–10.09)	0.87 (0.12–6.29)

Adjusted for age, income, body mass index status, systolic blood pressure, total cholesterol, fasting serum glucose, cigarette smoking, alcohol consumption, physical activity, medication use (aspirin, antihypertensive medication, antidiabetic medication, and statin), number of chronic diseases (hypertension, diabetes, and dyslipidemia), and Charlson Comorbidity Index at baseline.

* *p* value <0.05 and hazard ratio <1.

** *p* value <0.05 and hazard ratio >1.

A worsening smoking habit was defined as the current smoking status from non-smoking or past smoking, so cases of a worsening smoking habit can be classified as non-smoking or past smoking at baseline. Therefore, an additional analysis was conducted according to whether there was no smoking or past agreement at baseline (Table 3). For former smokers, the amount of smoking (pack*years) was corrected for analysis. Similar to the overall results, improvement in smoking habits showed a decrease in cardiovascular events and vice versa, an increase in cardiovascular events (Table 3).

**Table 3:** Association between dynamic changes in smoking habits and cardiovascular events according to the smoking status (never-smoker or past-smoker) before diagnosis and smoking amount.

Category	Mode1	Model 2	Model 3
	Multivariable-adjusted hazard ratio (95% CI)		
Worsening in smoking habit			
Men			
Myocardial infarct	2.13 (1.44–3.15)**	1.36 (0.75–2.45)	1.81 (1.30–2.51)**
Stroke	1.36 (1.05–1.77)**	1.30 (0.91–1.84)	1.34 (1.08–1.65)**
Cardiovascular death	1.58 (0.76–3.28)	3.48 (1.44–8.44)**	2.12 (1.22–3.68)**
Women			
Myocardial infarct	0.53 (0.07–3.75)	1.95 (0.22–16.93)	0.83 (0.20–3.38)
Stroke	1.99 (1.23–3.22)**	NA	1.85 (1.14–3.00)**
Cardiovascular death	1.10 (0.15–7.94)	NA	1.10 (0.15–7.91)
Improving in smoking habit			
Men			
Myocardial infarct			0.84 (0.65–1.09)
Stroke			0.78 (0.66–0.91)*
Cardiovascular death			0.49 (0.29–0.83)*
Women			
Myocardial infarct			1.56 (0.58–4.14)
Stroke			1.14 (0.66–1.98)
Cardiovascular death			2.13 (0.18–25.52)

Model 1: Adjusted for age, income, body mass index status, systolic blood pressure, total cholesterol, fasting serum glucose, cigarette smoking, alcohol consumption, physical activity, medication use (aspirin, antihypertensive medication, antidiabetic medication, and statin), number of chronic diseases (hypertension, diabetes, or dyslipidemia), Charlson Comorbidity Index at baseline in never smokers.

Model 2: Adjusted for age, income, body mass index status, systolic blood pressure, total cholesterol, fasting serum glucose, cigarette smoking, alcohol consumption, physical activity, medication use (aspirin, antihypertensive medication, antidiabetic medication, and statin), number of chronic diseases (hypertension, diabetes, or dyslipidemia), Charlson Comorbidity Index at baseline in past smokers.

Model 3: Adjusted for age, income, body mass index status, systolic blood pressure, total cholesterol, fasting serum glucose, cigarette smoking, alcohol consumption, physical activity, medication use (aspirin, antihypertensive medication, antidiabetic medication, and statin), number of chronic diseases (hypertension, diabetes, or dyslipidemia), Charlson Comorbidity Index at baseline and smoking amount (pack-years).

* *p* value < 0.05 and Hazard ratio <1.

** *p* value < 0.05 and Hazard ratio >1.

### Subgroup analysis

**Figure 2:**
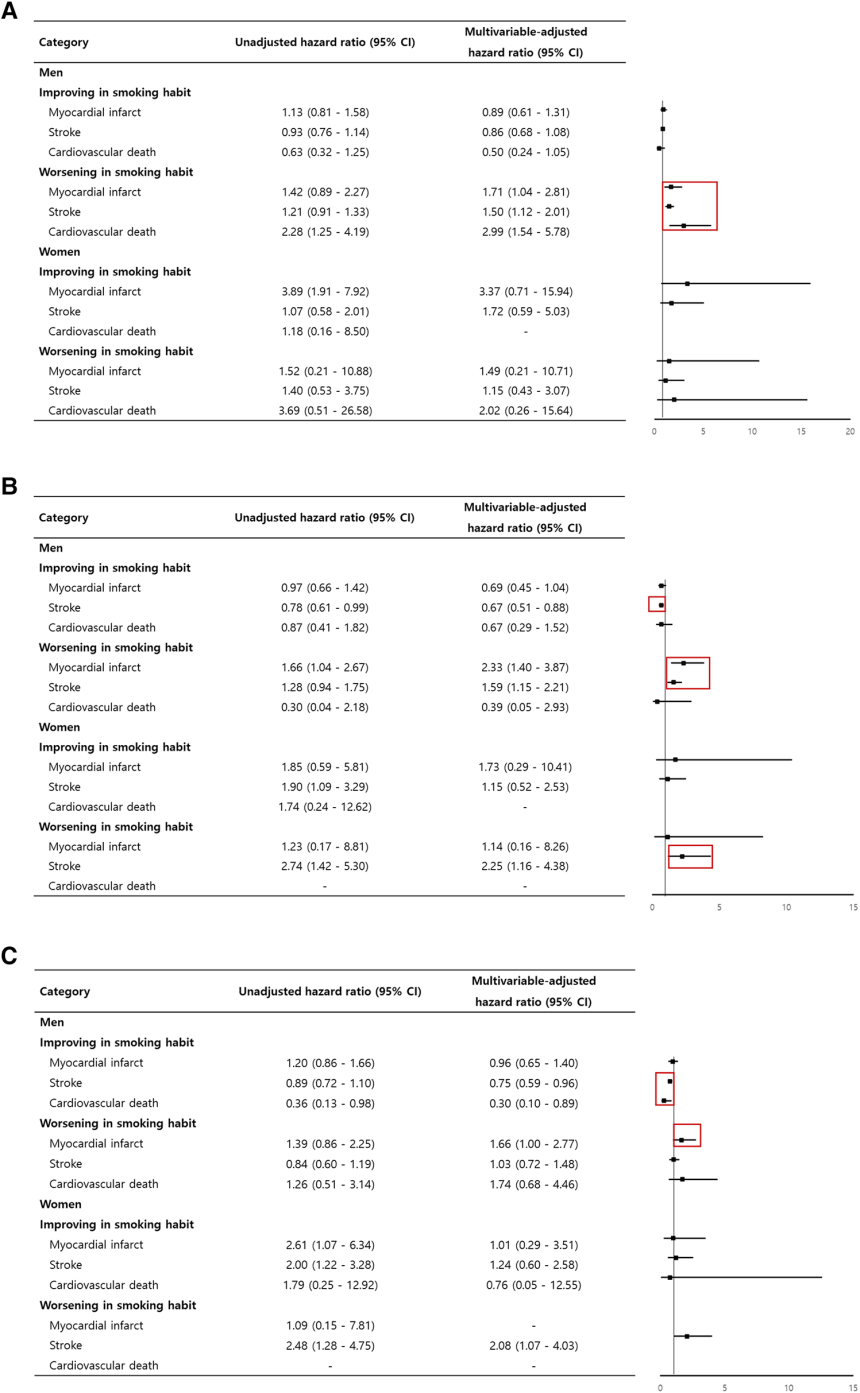
Adjusted hazard ratio for cardiovascular events according to dynamic changes in smoking habits in newly diagnosed (**A**) hypertension, (**B**) diabetes, (**C**) and dyslipidemia patients.

A newly diagnosed hypertension male, with worsening smoking status, had increased cardiovascular events. There was a trend in decreased cardiovascular events when the smoking habit was improved, but this was not statistically significant. In male patients newly diagnosed with diabetes, both the exacerbation and improvement of smoking habits were significantly associated with stroke risk. A similar trend was observed in women diagnosed with diabetes, and stroke increased when the smoking habit worsened. In men diagnosed with dyslipidemia, improvement in smoking habits significantly reduced stroke and cardiovascular death (Figure 2, Supplementary Tables S1–3).

## Discussion

This study found that compared with current smoking, smoking cessation among a newly-diagnosed disease population with higher cardiovascular risk with worsening smoking habit reaffirmed the cardiovascular benefit of smoking cessation demonstrated by others (8, 10, 11, 18, 19). It also revealed differences in statistical significance between sexes. Compared to non-smokers, the risk of stroke more than doubled in women with diabetes who started smoking in a relatively short period. The significance of this study is that it is real-world data showing the effect of changing smoking habits in patients newly diagnosed with hypertension, diabetes, or hyperlipidemia, even for a short period. Lifestyle modification for primary prevention is recommended as class I in several guidelines (AHA/ESC/ADA). The effect of lifestyle modification on diabetic patients was strongly recommended through the results obtained through randomized control and meta-analyses (20, 21).

In hypertensive patients, a randomized control trial showed that lifestyle modification was effective at preventing CVD by having a positive effect on blood pressure control. This has also been proven in several other studies, which also provided strong recommendations and high-quality evidence related to the guidelines (22–27).

In the real world, an improvement in everything in a positive direction may be limited, but other cohorts have shown that minimal improvement was sufficiently effective for cardiovascular adverse outcomes. It has been shown that the optimal correction of all aspects of lifestyle does not have a maximal effect; however, only minimal improvement is effective in low-risk subjects (28). Although this study was followed up for 10.9 years after diagnosis, it may be relatively short to confirm the cardiovascular adverse effects that can be obtained through the improvement of insulin resistance by improving exercise habits or weight control.

However, in subjects newly diagnosed with hypertension and diabetes during this period, a smoking-related lifestyle had a significant relationship with cardiovascular adverse events in newly diagnosed male patients with hypertension, and the worsening of smoking habit increased all-cause death, cardiovascular death, fatal and nonfatal myocardial infarct, and stroke. Also, in men and women diagnosed with diabetes, an increase in smoking significantly increased the risk of myocardial infarction and stroke.

A sub-analysis of overall lifestyle was also performed. For each of four items, smoking, exercise, weight control, and alcohol intake, improvements and deteriorations were evaluated (improvement plus 1, worsening minus 1), and a simple sum was calculated. If the value was positive, the overall lifestyle was seen to have improved. In this case, the relationship between lifestyle and cardiovascular events showed that the composite cardiovascular event increased in men (HR 1.16 95% CI 1.04–1.29) when the lifestyle worsened. In women, an improvement in overall lifestyle resulted in a 39% reduction in cardiovascular death (HR 0.61 95% CI 0.39–0.97). Generally, in women, overall lifestyle and especially smoking showed different associations with cardiovascular events. The reason may be that the number of analyzable numbers is small due to the small number of women who smoke.

We expected that overall lifestyle changes would lower the risk of cardiovascular events, but we did not observe such a trend. The reason can be explained as follows. In the evaluation of overall lifestyle, improvements were calculated by adding 1 point and deteriorations by subtracting 1 point. The proportion of each item involved in lifestyle improvement may vary from individual to individual. Therefore, evaluating improvement or deterioration by a simple summation method may be limited.

In the case of body weight, increases and decreases were not proportional to increases and decreases in the risk of cardiovascular events. Previous studies have demonstrated that weight gain had a protective effect on the cardiovascular system and that being slightly overweight rather than underweight had the lowest cardiovascular risk. For this reason, there are theories including lean body and muscle weight related to cardiovascular accidents that are not properly reflected in the body weight and obesity paradox (29, 30).

It is well known from previous studies that smoking contributes to an increased cardiovascular risk. These studies have demonstrated that smoking has adverse effects on cardiovascular events even when considering underlying conditions, race, and age. Conversely, it is also widely recognized that the opposite is true. Although the observation period for changes in smoking habits differs from study to study, the results of this study that worsening smoking habits in the short term after diagnosis, such as hypertension, diabetes, and hyperlipidemia, have negative effects on cardiovascular health are not only the subject of this study, but also more generally.

## Limitations

First, smoking status information was obtained through a self-reported survey, which is prone to reporting bias and potential misclassification. Secondly, the NHIS database lacked additional sources of information to assess cardiovascular outcomes, such as dietary factors and left ventricular ejection fraction. In addition, as the use of administrative databases becomes more common in clinical research, there is a potential for coding inaccuracies that may introduce errors into these studies. To mitigate this issue, we employed a previously validated definition based on previous studies that utilized a sample cohort from the Korean NHIS. Also, since our study relied on real-world data, we were unable to ascertain the specific reasons behind the participants’ lifestyle modifications. Another limitation is the small number of female smokers in the study, resulting in a relatively low rate of changes in smoking habits. Additionally, compared to men, women have a lower incidence of cardiovascular events. As a result, the occurrence of cardiovascular events based on changes in smoking habits did not show statistical significance or exhibited extremely wide 95% confidence intervals for incidence rates and hazard ratios. Finally, a limitation of this study is the restricted assessment of the duration of previous smoking and the period of cessation in past smokers. Therefore, further studies with different intervention strategies for lifestyle improvement to prevent cardiovascular events are needed.

## Conclusion

Our findings suggest that worsening smoking habits may increase the risk of myocardial infarction, stroke, and cardiovascular death in patients diagnosed with hypertension, diabetes, or dyslipidemia. Smoking is a risk factor for mortality and coronary heart disease in patients with hypertension, diabetes, or dyslipidemia, especially in men. Furthermore, the risk for stroke was more consistent in the hypertension male group and the newly diagnosed diabetes female group. For the primary prevention of cardiovascular events, lifestyle modifications should be actively considered.

## Data Availability

The datasets presented in this study can be found in online repositories. The names of the repository/repositories and accession number(s) can be found below: https://nhiss.nhis.or.kr.
