# Supplementary material for: Dynamic changing smoking habits and cardiovascular events in patients newly diagnosed with hypertension, diabetes, or dyslipidemia: a national cohort study
Source: Front Cardiovasc Med. 2023 Jun 28;10:1190227. doi: 10.3389/fcvm.2023.1190227 (PMC10336696; doi:10.3389/fcvm.2023.1190227)
Supplement: Supplementary file 1 [file Table1.docx]

Supplementary Table 1. The adjusted hazard ratio for cardiovascular events according to dynamic changes in smoking habits in newly diagnosed **hypertension** patients.

| Category | Number of participants | Cases | Incidence per 10,000 PY (95% CI) | Unadjusted hazard ratio (95% CI) | Multivariable-adjusted hazard ratio (95% CI) |
| --- | --- | --- | --- | --- | --- |
| Men |  |  |  |  |  |
| Improved smoking habit |  |  |  |  |  |
| Myocardial infarct | 1494 | 41 | 25.74  (18.96-34.96) | 1.13  (0.81-1.58) | 0.89  (0.61-1.31) |
| Stroke | 1494 | 110 | 70.19  (58.23-84.61) | 0.93  (0.76-1.14) | 0.86  (0.68-1.08) |
| Cardiovascular death | 1494 | 9 | 5.47  (2.85-10.51) | 0.63  (0.32-1.25) | 0.50  (0.24-1.05) |
| Worsening smoking habit |  |  |  |  |  |
| Myocardial infarct | 554 | 19 | 32.25  (20.57-50.56) | 1.42  (0.89-2.27) | 1.71 †  (1.04-2.81) |
| Stroke | 554 | 52 | 90.56  (69.00-118.84) | 1.21  (0.91-1.60) | 1.50 †  (1.12-2.02) |
| Cardiovascular death | 554 | 12 | 19.80  (11.25-34.87) | 2.28  (1.25-4.19) | 2.99 †  (1.54-5.78) |
| Women |  |  |  |  |  |
| Improved smoking habit |  |  |  |  |  |
| Myocardial infarct | 119 | 8 | 64.67  (32.34-129.32) | 3.89  (1.91-7.92) | 3.37  (0.71-15.94) |
| Stroke | 119 | 10 | 80.60  (43.37-149.80) | 1.07  (0.58-2.01) | 1.72  (0.59-5.03) |
| Cardiovascular death | 119 | 1 | 7.71  (1.09-54.77) | 1.18  (0.16-8.50) | NA |
| Worsening smoking habit |  |  |  |  |  |
| Myocardial infarct | 40 | 1 | 24.68  (3.48-175.21) | 1.52  (0.21-10.88) | 1.49  (0.21-10.71) |
| Stroke | 40 | 4 | 102.09  (38.32-272.02) | 1.40  (0.53-3.75) | 1.15  (0.43-3.07) |
| Cardiovascular death | 40 | 1 | 23.38  (3.29-166.00) | 3.69  (0.51-26.58) | 2.02  (0.26-15.64) |

Adjusted for age, income, body mass index status, systolic blood pressure, total cholesterol, fasting serum glucose, cigarette smoking, alcohol consumption, physical activity, medication use (aspirin and antihypertensive medication), and Charlson Comorbidity Index at baseline.

Supplementary Table 2. The adjusted hazard ratio for cardiovascular events according to dynamic changes in smoking habits in newly diagnosed **diabetes** patients.

| Category | Number of participants | Cases | Incidence per 10,000 PY | Unadjusted hazard ratio (95% CI) | Multivariable-adjusted hazard ratio (95% CI) |
| --- | --- | --- | --- | --- | --- |
| Men |  |  |  |  |  |
| Improved smoking habit |  |  |  |  |  |
| Myocardial infarct | 1105 | 31 | 26.33  (18.51-37.43) | 0.97  (0.66-1.42) | 0.69  (0.45-1.04) |
| Stroke | 1105 | 74 | 63.75  (50.76-80.06) | 0.78  (0.61-0.99) | 0.67 *  (0.51-0.88) |
| Cardiovascular death | 1105 | 8 | 6.61  (3.31-13.22) | 0.87  (0.41-1.82) | 0.67  (0.29-1.52) |
| Worsening smoking habit |  |  |  |  |  |
| Myocardial infarct | 394 | 19 | 45.15  (28.80-70.79) | 1.66  (1.04-2.67) | 2.33 †  (1.40-3.87) |
| Stroke | 394 | 43 | 104.70  (77.65-141.17) | 1.28  (0.94-1.75) | 1.59 †  (1.15-2.21) |
| Cardiovascular death | 394 | 1 | 2.30  (0.32-16.31) | 0.30  (0.04-2.18) | 0.39  (0.05-2.93) |
| Women |  |  |  |  |  |
| Improved smoking habit |  |  |  |  |  |
| Myocardial infarct | 85 | 3 | 34.44  (11.11-106.77) | 1.85  (0.59-5.81) | 1.73  (0.29-10.41) |
| Stroke | 85 | 13 | 153.83  (89.32-264.93) | 1.9  (1.09-3.29) | 1.15  (0.52-2.53) |
| Cardiovascular death | 85 | 1 | 10.96  (1.54-77.83) | 1.74  (0.24-12.62) | NA |
| Worsening smoking habit |  |  |  |  |  |
| Myocardial infarct | 42 | 1 | 22.89  (3.22-162.48) | 1.23  (0.17-8.81) | 1.14  (0.16-8.26) |
| Stroke | 42 | 9 | 220.71  (114.84-424.19) | 2.74  (1.42-5.30) | 2.25 †  (1.16-4.38) |
| Cardiovascular death | 42 | 0 | NA | NA | NA |

Adjusted for age, income, body mass index status, systolic blood pressure, total cholesterol, fasting serum glucose, cigarette smoking, alcohol consumption, physical activity, medication use (aspirin and antidiabetic medication), and Charlson Comorbidity Index at baseline.

Supplementary Table 3. The adjusted hazard ratio for cardiovascular events according to dynamic changes in smoking habits in newly diagnosed **dyslipidemia** patients.

| Category | Number of participants | Cases | Incidence per 10,000 PY | Unadjusted hazard ratio (95% CI) | Multivariable-adjusted hazard ratio (95% CI) |
| --- | --- | --- | --- | --- | --- |
| Men |  |  |  |  |  |
| Improved smoking habit |  |  |  |  |  |
| Myocardial infarct | 1571 | 44 | 26.25  (19.54-35.28) | 1.20  (0.86-1.66) | 0.96  (0.65-1.40) |
| Stroke | 1571 | 102 | 61.55  (50.70-74.74) | 0.89  (0.72-1.10) | 0.75 *  (0.59-0.96) |
| Cardiovascular death | 1571 | 4 | 2.32  (0.87-6.19) | 0.36  (0.13-0.98) | 0.30 *  (0.10-0.89) |
| Worsening smoking habit |  |  |  |  |  |
| Myocardial infarct | 552 | 18 | 30.48  (19.20-48.37) | 1.39  (0.86-2.25) | 1.66  (1.00-2.77) |
| Stroke | 552 | 34 | 58.25  (41.62-81.53) | 0.84  (0.60-1.19) | 1.03  (0.72-1.48) |
| Cardiovascular death | 552 | 5 | 8.24  (3.43-19.80) | 1.26  (0.51-3.14) | 1.74  (0.68-4.46) |
| Women |  |  |  |  |  |
| Improved smoking habit |  |  |  |  |  |
| Myocardial infarct | 122 | 5 | 39.00  (16.23-93.70) | 2.61  (1.07-6.34) | 1.01  (0.29-3.51) |
| Stroke | 122 | 16 | 127.50  (78.11-208.11) | 2.00  (1.22-3.28) | 1.24  (0.60-2.58) |
| Cardiovascular death | 122 | 1 | 7.54  (1.06-53.50) | 1.79  (0.25-12.92) | 0.76  (0.05-12.55) |
| Worsening smoking habit |  |  |  |  |  |
| Myocardial infarct | 57 | 1 | 16.42  (2.31-116.58) | 1.09  (0.15-7.81) | NA |
| Stroke | 57 | 9 | 156.36  (81.36-300.51) | 2.46  (1.28-4.75) | 2.08 †  (1.07-4.03) |
| Cardiovascular death | 57 | 0 | NA | NA | NA |

Adjusted for age, income, body mass index status, systolic blood pressure, total cholesterol, fasting serum glucose, cigarette smoking, alcohol consumption, physical activity, medication use (aspirin and statin), and Charlson Comorbidity Index at baseline.
